# Cost-effectiveness of remdesivir for the treatment of hospitalized patients with COVID-19: a systematic review

**DOI:** 10.1186/s40249-023-01092-1

**Published:** 2023-04-20

**Authors:** Aziz Rezapour, Zahra Behroozi, Mostafa Nasirzadeh, Mohsen Rezaeian, Mohammad Barzegar, Mahsa Tashakori-Miyanroudi, Abdollah Sayyad, Aghdas Souresrafil

**Affiliations:** 1 Health Management and Economics Research Center, Health Management Research Institute, Iran University of Medical Sciences, Tehran, Iran; 2 Physiology Research Center, Institute of Neuropharmacology, Kerman University of Medical Sciences, Kerman, Iran; 3 Department of Health Education and Health Promotion, School of Health, Occupational Environment Research Center, Rafsanjan University of Medical Sciences, Rafsanjan, Iran; 4 Department of Epidemiology and Biostatistics, School of Medicine, Occupational Environment Research Center, Rafsanjan University of Medical Sciences, Rafsanjan, Iran; 5 Department of English Language Teaching, School of Health Management and Information Sciences, Iran University of Medical Sciences, Tehran, Iran; 6 Psychiatry and Behavioral Sciences Research Center, Addiction Institute, Mazandaran University of Medical Sciences, Sari, Iran; 7 Department of Health Services and Health Promotion, School of Health, Occupational Environment Research Center, Rafsanjan University of Medical Sciences, Rafsanjan, Iran

**Keywords:** Cost-effectiveness, Remdesivir, COVID-19, SARS-CoV-2

## Abstract

**Background:**

Remdesivir is being studied and used to treat coronavirus disease 2019 (COVID-19). This study aimed to systematically identify, critically evaluate, and summarize the findings of the studies on the cost-effectiveness of remdesivir in the treatment of hospitalized patients with COVID-19.

**Methods:**

In this systematic review, PubMed, EMBASE, Web of Science, SCOPUS, and the Cochrane Library were searched for studies published between 2019 and 2022. We included all full economic evaluations of remdesivir for the treatment of hospitalized patients with COVID-19. Data were summarized in a structured and narrative manner.

**Results:**

Out of 616 articles obtained in this literature search, 12 studies were included in the final analysis. The mean score of the Quality of Health Economic Studies (QHES) for the studies was 87.66 (high quality). All studies were conducted in high-income countries (eight studies in the USA and one study in England), except for three studies from middle-to-high-income countries (China, South Africa, and Turkey). Six studies conducted their economic analysis in terms of a health system perspective; five studies conducted their economic analysis from a payer perspective; three studies from the perspective of a health care provider. The results of five studies showed that remdesivir was cost-effective compared to standard treatment. Furthermore, the therapeutic strategy of combining remdesivir with baricitinib was cost-effective compared to remdesivir alone.

**Conclusions:**

Based on the results of the present study, remdesivir appears to be cost-effective in comparison with the standard of care in China, Turkey, and South Africa. Studies conducted in the United States show conflicting results, and combining remdesivir with baricitinib is cost-effective compared with remdesivir alone. However, the cost-effectiveness of remdesivir in low-income countries remains unknown. Thus, more studies in different countries are required to determine the cost-effectiveness of this drug.

**Supplementary Information:**

The online version contains supplementary material available at 10.1186/s40249-023-01092-1.

## Background

For the first time in December 2019, the fatal coronavirus disease 2019 (henceforth referred to as COVID-19) as a respiratory disorder commenced and is globally expanding [1]. It seems it is the greatest global public health crisis after the influenza pandemic in 1918 [2]. The World Health Organization (WHO) reported that over 574 million people contracted COVID-19 and over 6.3 million of them died worldwide as of 31 July 2022 [3]. A study indicated that among 20 countries with the highest disability-adjusted life years (DALYs), the top five countries with the highest losses due to COVID-19 included the USA, Brazil, Italy, Mexico, and India [2].

Inhibition and treatment of COVID-19 can be very expensive. Due to the high contagiousness of COVID-19, the demand for healthcare has nearly outpaced supply. According to the first wave, admissions to the intensive care unit increased by 12% in Italy and by 236% in the UK [4–6]. From January to March 2020, the health care cost of the COVID-19 outbreak in China was estimated to be USD 0.62 billion (an average of USD 939 for non-severe patients and USD 25,578 for severe patients), and the societal cost associated with the COVID-19 outbreak was USD 383 billion [7].

According to recent studies, patients with severe COVID-19 often utilize mechanical ventilation and extracorporeal membrane oxygenation (ECMO) which are very expensive medical procedures and can significantly place a substantial economic burden on the healthcare system [8, 9].

In addition to clinical care and oxygen, a limited number of drugs have hitherto been used to alleviate COVID-19 complications, but most of them have not been useful [10]. A number of pharmaceutical treatments such as Hydroxychloroquine, remdesivir, cacirivimab-imidumab, dexamethasone, baricitinib-remdesivir, Tocilizumab, lopinavir-ritonavir-1, and interferon are available for COVID-19 hospitalized patients, with randomized controlled trials (RCTs) or meta-analyses [11]. The antiviral Remdesivir (GS-5734) works by inhibiting replication of the SARS-CoV-2 virus’s genome through RNA-dependent RNA polymerase (RdRp)[12]. For this reason, it has been known as a promising therapy for COVID-19. Studies have indicated that remdesivir therapy for COVID-19 is effective, but it seems relatively expensive in competition with other COVID-19 treatments [13–15]. For example, in a study conducted in the United States, remdesivir treatment cost approximately USD 13,000 per patient with COVID-19, and the incremental cost compared to other treatment approaches was reported to be USD 2,000 per patient [14].

Systematic reviews are a suitable tool that analyzes clinical evidence and helps the improvement of practical guidance and correct medical decisions [16]. Because of the importance of economic evaluations of remdesivir as the first treatment in decreasing the symptoms of COVID-19, the present review aimed to review the existing literature about the cost-effectiveness of remdesivir in comparison with other treatments.

## Methods

The present study was conducted in accordance with Additional file 1: PRISMA (Preferred Reporting Items for Systematic Reviews and Meta-Analyses) guidelines and Additional file 2: AMSTAR (Assessing the Methodological Quality of Systematic Reviews) guidelines [17, 18]. The study protocol was registered in PROSPERO (IDCRD42022359040).

### Data sources

A literature search on the cost-effectiveness of remdesivir in patients with COVID-19 was conducted in PubMed, Scopus, EMBASE, Web of Science, and Cochrane databases from December 2019 to May 2022. Moreover, the search engine ‘Google Scholar’ was searched to find theses and organizational reports of economic evaluations.

### Search strategy

The electronic search strategy was based on patients (COVID-19), intervention (remdesivir), and outcomes (cost, cost-effectiveness, cost-utility, and cost-benefit) in different spellings (Additional file 3: Table S1). The reference lists of the included studies were also searched for additional studies.

### Study selection

All identified studies were transferred to EndNote software X7 (Thomson Reuters, New York, USA), and duplicate studies were removed. Afterward, reviewer 1 (AS) screened the titles and abstracts. Subsequently, the full texts of the studies were screened based on inclusion and exclusion criteria. Studies on full economic evaluation were included in the final review if they fulfilled all of the following criteria: The study population was hospitalized patients with COVID-19, and remdesivir was a treatment option in the study. The comparator used in the study was any other drugs, and the study reported the results of full economic evaluations, including the cost-effectiveness ratio. On the other hand, the following types of studies were not eligible: partial evaluations, cost-of-illness studies, conference abstracts, comments, letters, editorials, and preprints. Additionally, studies with unclear treatment options or those that did not accurately report the cost and effectiveness of each treatment were excluded from the present study.

### Data extraction

Data extraction forms were created from priori, and they included five fields: general information, characteristics of studied patients and interventions, details of methods, and economic evaluation results. Two reviewers (AS and AR) extracted the data independently.

### Quality assessment of the studies

In order to assess the quality of cost-effectiveness analysis, we chose the Quality of Health Economics Studies (QHES) tool [19]. We used this tool because it specifically addresses questions about the quality of the studies on health economic analyses. Using this checklist, two of the present researchers independently evaluated the quality of the studies, and they resolved their disagreements by discussion. We calculated the mean total score of QHES, standard deviation, and minimum and maximum values for all studies. Studies were categorized into four quartiles based on their quality: very poor quality (0–24), low quality (25–49), fair quality (50–74), and high quality (75–100) [20]. The results of the data extraction from the articles were discussed and analyzed using qualitative synthesis methods after assessing the quality of the methodology.

## Results

**Fig. 1:**
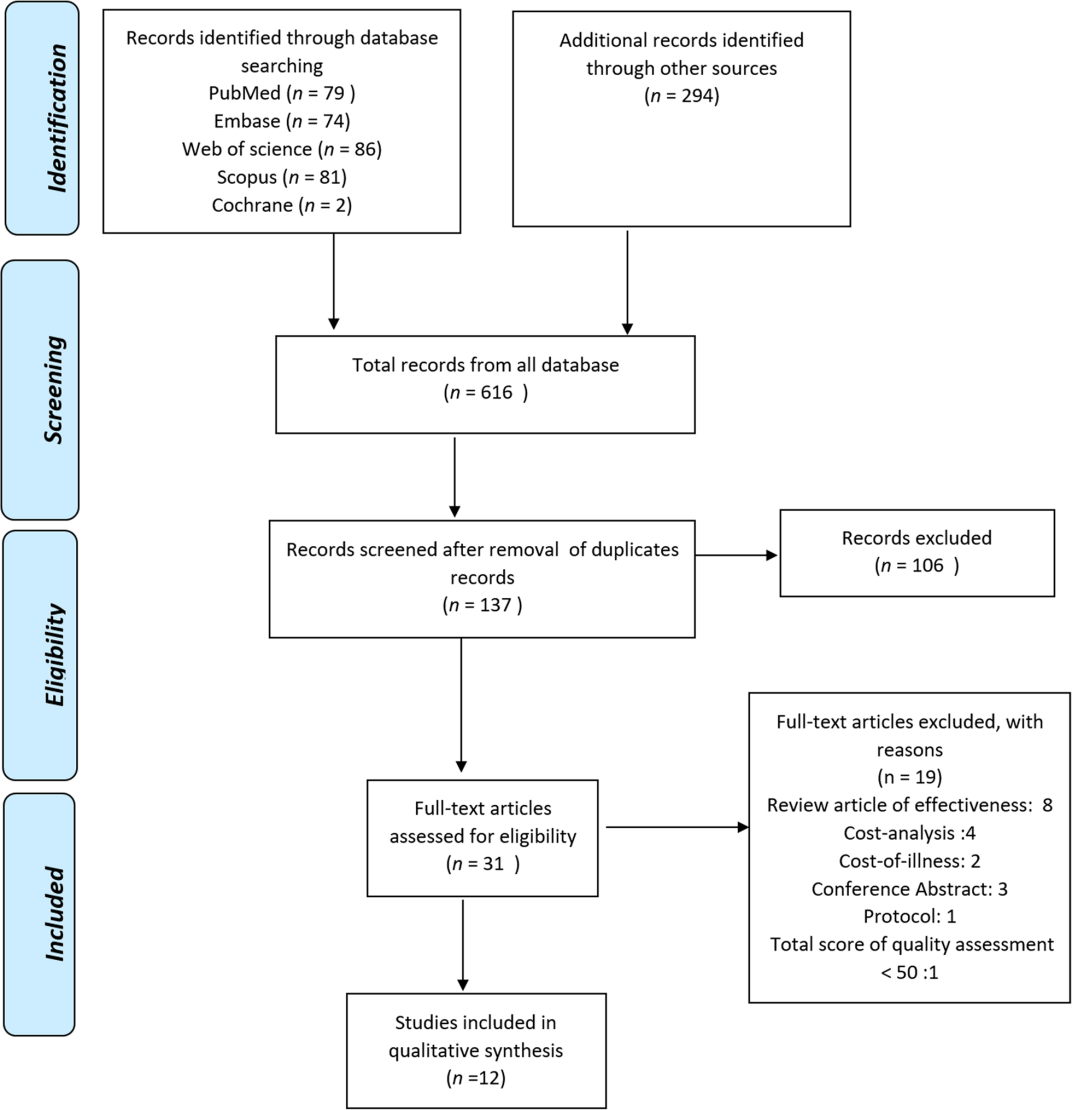
PRISMA flow chart for study selection. *PRISMA* Preferred Reporting Items for Systematic Reviews and Meta-Analyses

A total of 616 potentially relevant articles were identified, 12 of which met the inclusion criteria. (Fig. 1) [11, 14, 15, 21–29]. The characteristics of these studies are described in Table 1. All studies were conducted in high-income countries (eight studies in the USA [11, 14, 21, 24, 25, 27–29] and one study in England [26]) except for three studies from upper-middle-income countries (China, South Africa, and Turkey) [15, 22, 23]. Of the 12 articles in this review, one study included cost-effectiveness analysis (CEA) [23], 10 studies included cost-utility analysis (CUA) [11, 14, 15, 21, 22, 24, 26–29], and two studies included both CEA and CUA [25, 27]. Six studies conducted their economic analysis from the perspective of a health system [11, 21–23, 27, 28], five studies from a payer perspective [14, 15, 24–26], and three studies from the perspective of a health care provider [24, 25, 29].

**Table 1:** The characteristics of included studies in the present review

Author name (year of publication)	Setting	Comparators	Population	Type of economic evaluation(model)	Perspective	Time horizon	Discount rate(%)	Sensitivity analyses	Costing year
Whittington (2022)	US	- Remdesivir + SoC (dexamethasone) - SoC	Hospitalized patients with COVID-19	CUA (Markov)	US healthcare sector	Lifetime	3	Yes, one-way, two-way and PSA	2020
Rafia (2022)	England and wales	- Remdesivir - SoC	Hospitalized patients with COVID-19	CUA (Decision –analysis model)	NHS/personal social services (payer)	Lifetime	3.5	Yes, DSA and PSA	NR
Kelton (2022)	US	-Baricitinib + Remdesivir - Remdesivir	Hospitalized patients with COVID-19	CUA (Markov)	Payer and hospital	Lifetime	3	Yes, DSA and PSA	NR
Dijk (2022)	US	- Hydroxychloroquine - Remdesivir - Casirivimab-Imdevimab - Dexamethasone - Baricitinib + Remdesivir - Tocilizumab - Lopinavir-ritonavir - Interferon beta-1a - Usual care	Hospitalized patients with COVID-19	CUA (state-transition model )	US health care	Lifetime	3	Yes, PSA	2020
Jo (2021)	South Africa	(1) Remdesivir administered to nonventilated patients and dexamethasone administered to ventilated patients, (2) dexamethasone alone administered to both nonventilated and ventilated patients, (3) Remdesivir administered to nonventilated patients only (4) dexamethasone administered to ventilated patients only, all relative to standard of ICU care	COVID patients	CEA	Health care system	Between august 2020 and January 2021	5 for the capital asset (e.g. ventilators)	Yes, One-way, 3-way and PSA	2020
Jiang (2021)	-Remdesivir-SoC	Sever COVID-19 patients	CUA (dynamic compartment transmission model)	Health care system	55-day	NA	Yes, One-way and PSA	2020	
Okuz (2021)	Turkey	- Remdesivir - SoC	COVID-19 patients hospitalized with ≤ 94% saturation and low-flow oxygen therapy requirement.	CUA (cost-effectiveness model)	Payer	A COVID-19 episode time	NA	Yes, PSA	2020
Ohsfeldt (2021)	US	- Baricitinib + SOC - SOC alone (which included systemic corticosteroids and Remdesivir)	Hospitalized patients with COVID-19	CEA and CUA (Markov)	Third-payer and hospital	Lifetime	3	Yes, one-way and PSA	2020
Congly (2021)	US	Supportive care, Dexamethasone severe, Dexamethasone all, Remdesivir severe, Remdesivir moderate, Remdesivir moderate, dexamethasone severe, Remdesivir all	Moderate to severe COVID-19 patients	CUA (decision tree)	Payer	One year	NA	Yes, univariate and PSA	2020
Carta (2021)	US	- SoC - Dexamethasone - Remdesivir - Remdesivir + dexamethasone	Hospitalized COVID-19 patients	CUA (decision tree)	Health care system	One year	NA	Yes, One-way and PSA	NR
Wu (2021)	US	Remdesivir + SoC vs. SoC	Hospitalized, adult patient with COVID-19	CUA (Decision tree)	Provider (hospital)	30 days	NA	No	2020
Whittington (2020)	US	- Remdesivir plus SoC - SoC	Hospitalizes patients with COVID-19	CEA and CUA (decision tree and Markov)	Health care system	Lifetime	3	Yes, scenario analysis	2020

*SoC* Standard of care, *CEA* Cost-effectiveness analysis, *CUA* Cost-utility analysis, *DSA* Deterministic sensitivity analysis, *PSA* Probabilistic sensitivity analysis, *NA* Not applicable, *NR* Not reported, Supportive care: The SC was taken into consideration for patients who received neither therapy as a comparator

The costing year for three studies was not reported [21, 24, 26]. The time frames or study horizons adopted in the studies ranged from 30 days to a lifetime: three studies less than 1 year [22, 23, 29]; two studies one year [14, 21]; 6 studies a lifetime [11, 24–28]. In addition, one study was conducted in horizon time of a COVID-19 episode [15]. In the studies using discount rates, annual rates ranged from 3 to 5%.

The cost-effectiveness analysis in the US used willingness-to-pay (WTP) thresholds below USD 50,000 and USD 100,000 [11, 14, 22, 24, 25, 28]. The WTP threshold accepted by a Chinese study was USD 10,276 per QALY [22]. In England, Turkey, and South Africa, the WTP thresholds were GBP 20,000 per QALY, USD 8599 and USD 25,797 per QALY, and USD 3015 per DALY avoided and USD 36,000 per death avoided, respectively [15, 23, 26].

### Results of quality assessment of economic evaluation studies

**Fig. 2:**
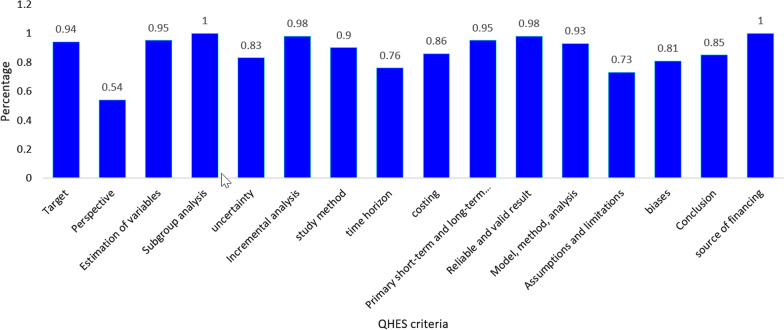
Results of quality assessment of the methodology of studies using the QHES checklist. *QHES* Quality of Health Economic Studies

The mean QHES score of the studies was 87.66. The maximum and minimum scores were 96 and 74.5, respectively (Table 2). All studies, except for one study [28], received a high-quality total score (total score < 75). The bar graph in Fig. 2 shows the percentage score for each criterion that all studies obtained. The total score in the criteria of subgroup analysis and the report of the funding source of the study were obtained for 100% of economic evaluations.

**Table 2:** Result of quality assessment of included studies

Criteria of QHES	Whittington (2022)	Rafia (2022)	Kelton (2022)	Dijk (2022)	Jo (2021)	Jiang (2021)	Okuz (2021)	Ohsfeldt (2021)	Congly (2021)	Carta (2021)	Wu (2021)	Whittington (2020)
Target	6	7	7	7	7	6	6	7	6	7	6	7
Perspective	2	4	2	2	2	2	2	2	2	2	2	2
Estimation of variables	8	8	8	8	4	8	8	8	8	8	8	8
Subgroup analysis	1	1	1	1	1	1	1	1	1	1	1	1
uncertainty	4.5	9	9	4.5	9	9	4.5	9	9	9	4.5	9
Incremental analysis	6	6	6	6	6	6	6	5	6	6	6	6
study method	4	5	5	5	5	4	4	5	4	4	4	5
time horizon	7	7	7	7	3	4	3	7	5	5	3	6
costing	6	7	8	7	8	8	6	8	6	6	5	8
Primary short-term and long-term outcomes	5	6	6	6	6	6	5	6	5	6	6	6
Reliable and valid result	6	7	7	7	7	7	7	7	7	7	7	7
Model, method, analysis	6	8	8	8	8	8	4	8	8	8	8	8
Assumptions and limitations	4	6	4	4	6	6	5	4	6	6	6	5
biases	2	6	4	5	6	5	5	5	5	5	5	6
Conclusion	4	6	6	6	6	8	8	6	8	8	8	8
source of financing	3	3	3	3	3	3	3	3	3	3	3	3
Total score	74.5	96	91	86.5	87	91	77.5	91	89	91	82.5	95

### Cost-effectiveness results of remdesivir compared with the standard of care

The costs and outcomes of the interventions are shown in Table 3. The results of an American study showed that considering the current price of remdesivir (USD 520 per vial) and the hypothesis of no effect of remdesivir on survival, this drug was not cost-effective in mild to severe COVID-19 patients. The incremental cost-effectiveness ratios (ICER) in this study for moderate to severe and mild COVID-19 patients were reported as USD 298,200 and USD 1,847,000 per QALY, respectively. The results of the deterministic sensitivity analysis in this study showed that three variables, including the survival related to remdesivir, the probability of death among patients receiving standard treatment, and the hospitalization cost of COVID-19 patients, affected the results of the study. In addition, the cost-effectiveness results varied with the change in the risk of death among patients treated with the standard of care [28]. The results of a study carried out in the UK showed that the use of remdesivir was cost-effective compared to the standard of care for the treatment of hospitalized patients with COVID-19, assuming the effect of remdesivir on reducing mortality (Cost-effectiveness ratio = GBP 11,881 per QALY). The results of probabilistic sensitivity analysis showed that remdesivir was cost-effective compared to standard treatment with a probability of 0.74. Furthermore, the results of the threshold analysis in this study indicated that when the survival risk ratio of remdesivir is greater than 0.915 (i.e. when no difference in survival between remdesivir and standard treatment is assumed), the price of each 100-mg vial of remdesivir should be less than GBP 18.6 so that remdesivir remains cost-effective. When assumed that remdesivir has no effect on reducing the mortality rate, it will have a small effect on increasing the quality of life. Moreover, 52% of the points of the probability simulation results will be in the northern quadrant (higher cost and greater effectiveness), and remdesivir will not be cost-effective at GBP 20,000 anymore. Furthermore, evidence showed that patients who require low-flow oxygen (LFO) are more likely to benefit from remdesivir compared to patients who require high-flow oxygen (HFO) or noninvasive ventilation (NIV) [26, 30]. Therefore, the administration of remdesivir to patients with LFO is cost-effective [26]. According to a study performed in Turkey, remdesivir use reduces hospitalization by 3 days compared with standard care. Low requirements for ventilators in treatment with remdesivir caused an increase in QALY compared to the standard of care. The difference in QALY in the investigated groups was estimated to be 0.174. The average cost of an episode for each patient in the remdesivir arm was USD 34,611.1, and in the standard of care arm, it was USD 3538.9. Remdesivir, compared to the standard of care, led to a shorter length of stay and less need for intubation among intensive care unit patients, and in patients with spo2 < 94%, who required oxygen support, it was the dominant option (higher QALY and lower costs) [15].

**Table 3:** Main cost-effectiveness results

Reference (year of publication)	Intervention				Total Costs (USD 2020)	Mean QALY/ QALD/ Disutility / Death averted/ evLYG						Cost-effectiveness measure(USD 2020)	Cost-effective against the standard of care?
						QALY	LYG	QALD	Disutility	Death averted	evLYG		
Whittington (2022)	Moderate to severe population		SoC + Remdesivir		313,447	12.189	–	–	–	–	–	Incremental Cost per QALY: USD 298, 200 Value-based price : 4000–13,500	No
			SoC		311,617	12.182	–	–	–	–	–	Ref.	
	Mild population		SoC + Remdesivir		318,375	13.702	–	–	–	–	–	Incremental Cost per QALY: 1,847, 000 Value-based price : 700-4,600	
			SoC		315,629	13.701	–	–	–	–	–	Ref.	
Rafia (2022)	SoC				12,958.40	6.35	–	–	–	–	–	Ref.	It is cost-effective if remdesivir prevents death
	Remdesivir				17,558.59	6.63	–	–	–	–	–	Incremental Cost per QALY: 16,403.02	
Kelto (2022)	Payer perspective	All patient		Baricitinib-Remdesivir	380,396	12.0641	15.0641	–	–	–	–	Incremental Cost per QALY: 22,334 Incremental Cost per LYG:17,858	Baricitinib-Remdesivir is cost-effective compared to using REM for hospitalized patients with COVID-19.
				Remdesivir	372,434	11.7076	14.754	–	–	–	–	Ref.	
		Patient with oxygen use		Baricitinib-Remdesivir	379,487	11.9480	15.056	–	–	–	–	Incremental Cost per QALY: 21,818 Incremental Cost per LYG:17,458	
				Remdesivir	370,527	11.5373	14.543	–	–	–	–	Ref.	
	Hospital perspective	All patient		Baricitinib-Remdesivir	Incremental total expenditure : − 1778	Incremental total QALYs: 0.0018	–	–	–	–	–	Dominates	
				Remdesivir			–	–	–	–	–	Ref.	
		Patient with oxygen use		Baricitinib-Remdesivir	Incremental total expenditure : − 2709	Incremental total QALYs: 0.0023	–	–	–	–	–	Dominates	
				Remdesivir			–	–	–	–	–	Ref.	
Dijk (2022)	Casirivimab-Imdevimab				Incremental cost : 696	Incremental QALY : 0.171	–	–	–	–	–	Incremental Cost per QALY: 4075 ICER< WTP	Yes
	Remdesivir				Incremental cost : − 5	Incremental QALY : 0.252	–	–	–	–	–	Dominates	
	Dexamethasone				Incremental cost :6856	Incremental QALY : 0.614	–	–	–	–	–	Incremental Cost per QALY: 111,699 ICER< WTP	
	Baricitinib-Remdesivir				Incremental cost : 10,673	Incremental QALY : 0.775	–	–	–	–	–	Incremental Cost per QALY: 13,772 ICER< WTP	
	Tocilizumab				Incremental cost : 35,849	Incremental QALY : 0.882	–	–	–	–	–	Incremental Cost per QALY: 40,633 ICER< WTP	
	Interferon beta-1a				Incremental cost : − 2538	Incremental QALY :− 0.472	–	–	–	–	–	Incremental Cost per QALY: 5377 Cost-saving	
	Lopinavir-ritonavir				Incremental cost : − 1404	Incremental QALY : − 0.091	–	–	–	–	–	Incremental Cost per QALY:15418 Cost-saving	
	Hydroxychloroquine				Incremental cost : − 12,227	Incremental QALY :− 0.263	–	–	–	–	–	Incremental Cost per QALY:46427 Cost-saving	
Jo (2021)	SoC				83,937	–	–	–	–	Ref.	–	Ref	
	Remdesivir for non-ventilated patients & dexamethasone for ventilated patients				69,346	–	–	–	–	408	–	Incremental Cost per death averted: Cost-saving	Yes, the use of remdesivir for nonventilated patients and dexamethasone for ventilated patients is likely to be cost-saving.
	Dexamethasone for non-ventilated & ventilated patients				84,096	–	–	–	–	689	–	Incremental Cost per death averted: USD 231	
	Remdesivir for non-ventilated patients (58%) only				69,279	–	–	–	–	26	–	Incremental Cost per death averted: Cost-saving	
	Dexamethasone for ventilated patients (42%) only				84,003	–	–	–	–	382	–	Incremental Cost per death averted: USD174	
Jiang (2021)	SoC				641,356,115.72	–	–	512,651,191	–	–	–	Ref	Yes
	Remdesivir				669,201,230.38	–	–	512,677,159	–	–	–	ICER: USD 4,008.53	
Okuz (2021)	SoC				3538.9	–	–	–	0.515	–	–	INB: USD 1575.6 (1*GDP) INB: USD 4,571.4 (3*GDP)	Yes
	Remdesivir				3461.1	–	–	–	0.341	–	–		
Ohsfeldt (2021)	Payer perspective	Placebo + SoC(systemic corticosteroids and Remdesivir)			329,268	11.3879	14.300	–	–	–	–	Ref.	Addition of baricitinib to SOC (systemic corticosteroids and Remdesivir) was cost-effective.
		Baricitinib + SoC			346,544	12.0582	15.137	–	–	–	–	Incremental Cost per QALY: USD 25,774 Incremental Cost per LYG: USD 20,638	
	Hospital perspective	Placebo + SoC(systemic corticosteroids and Remdesivir)			Incremental total expenditure: − 2436	Incremental total QALYs: 0.0023	–	–	–	–	–	Ref.	
		Baricitinib + SoC					–	–	–	–	–	Dominates	
Congly (2021)	Supportive care				11,112.98	0.7155	–	–	–	–	–	–	No
	Dexamethasone severe				11,115.86	0.7256	–	–	–	–	–	–	
	Dexamethasone all				11,132.18	0.7351	–	–	–	–	–	–	
	Remdesivir severe				11,756.48	0.7100	–	–	–	–	–	–	
	Remdesivir moderate				13,101.98	0.7245	–	–	–	–	–	–	
	Remdesivir moderate, dexamethasone severe				13,104.86	0.7346	–	–	–	–	–	–	
	Remdesivir all				13,745.48	0.7190	–	–	–	–	–	–	
Carta (2021)	SoC				33,369.9	0.7673	–	–	–	–	–	–	Yes
	dexamethasone				33,555.6	0.803	–	–	–	–	–	–	
	Remdesivir				32,354.4	0.7734	–	–	–	–	–	Dominant	
	Remdesivir+ dexamethasone				32,540.2	0.809	–	–	–	–	–	Dominant	
Wu (2021)	SoC				31,159	0.04376	–	–	–	–	–	ICER: USD 32,809,252	No
	SoC + Remdesivir				36,990	0.04554	–	–	–	–	–		
Whittington (2020)	SoC				305,230	12.18	–	–	–	–	12.19	Ref	This study aimed to provide pricing estimates for remdesivir for the treatment of COVID-19.
	SoC + Remdesivir				319,230	12.46	–	–	–	–	12.48	WTP = USD50,000 per QALY and per evLYG Cost-effectiveness price benchmark: USD 4580–USD 5080 WTP = USD100,000 per QALY and per evLYG, Cost-effectiveness price benchmark: USD 18,640 - USD 19,630 WTP = USD 150,000 per QALY and per evLYG, Cost-effectiveness price benchmark: USD 32,700–USD 34,180	

*SoC* Standard of care, *QALY* Quality adjusted life-years, *evLYG* Equal value of life years gained, *QALD* Quality adjusted life-days, *LYG* Life years gained, *WTP* Willingness-to-pay, –: Not applicable

In a study in China, the cost-effectiveness of 5-day remdesivir compared with the standard of care was examined among severe COVID-19 patients. The results of this study showed that the cost of treatment with remdesivir was CNY 97.93 million more than the standard of care. The ICER was CNY 14,098 per QALY, which was lower than China’s willingness-to-pay threshold. Daily severe cases of COVID-19 were 19% lower in the remdesivir treatment strategy than in the standard of care. The results of the study were robust to changes in the severity of the epidemic, modelling methods, and most of the model parameters. However, the results were relatively sensitive to changes in efficacy estimates [22]. Another study was conducted to investigate the cost-effectiveness of remdesivir compared to standard treatment with the decision tree technique from the perspective of the hospital. In this study, the ICER of the combination of remdesivir and the standard of care compared to the standard of care was estimated at USD 346,622, and remdesivir was not recognized as a cost-effective drug [29]. In an American study comparing the cost-effectiveness of pharmacological treatments for COVID-19, it was concluded that such drugs as remdesivir, casirivimab, imdevimab, dexamethasone, Baricitinib, and Tocilizumab were cost-effective compared to usual care, while treatments with Hydroxychloroquine, lopinavir-ritonavir, and interferon beta-1a were not cost-effective. Remdesivir was a dominant drug (lower cost and more effective) compared to conventional treatments, and the ICER of other drugs (casirivimab, imdevimab, etc.) was below the willingness-to-pay threshold, and they were cost-effective [11]. A study conducted in South Africa investigated the cost-effectiveness of remdesivir compared to the standard of care in non-ventilated patients and the cost-effectiveness of dexamethasone for non-ventilated and ventilated patients. The results of this study showed that remdesivir in non-ventilated patients and dexamethasone in ventilated patients prevented 408 deaths and saved 15 million dollars in costs. This result was due to the effectiveness of dexamethasone and the reduction of the required time in the ICU for patients treated with remdesivir. Compared to the standard of care, the use of remdesivir for non-ventilated patients and dexamethasone for ventilated patients would probably result in cost savings by reducing the ICU length of stay. Much uncertainty was observed in reducing the effectiveness and length of stay of remdesivir. Drug cost, cost per day of stay in the ICU, and mortality rate in the ICU were the most important influencing factors in the analysis of dexamethasone sensitivity in non-ventilated and ventilated patients [23].

In a study with a time horizon of one year and from the payer perspective in the United States, COVID-19 patients who required intubation and intensive care were classified as patients with severe COVID-19 and those requiring oxygen as patients with moderately severe COVID-19. Treatment strategies considered in this study included remdesivir for all patients, remdesivir for patients with only moderate and only severe infections, dexamethasone for all patients, dexamethasone for severe infections, remdesivir for moderate infections/dexamethasone for severe infections, and best supportive care. The results of this study showed that the use of dexamethasone was the most cost-effective strategy for all patients, with an ICER of USD 980.84 per QALY. However, the remdesivir treatment strategy was more expensive and less effective than other strategies. Dexamethasone was cost-effective for all patients in 98.3% of scenarios. Dexamethasone was the most cost-effective strategy for moderately severe infections. On the basis of the current data, remdesivir was unlikely to be a cost-effective treatment for COVID-19. The results of sensitivity analysis in this study showed that dexamethasone was cost-effective for all patients when the willingness-to-pay threshold was more than USD 1250 per QALY [14]. A study in the USA examined the cost-effectiveness of remdesivir and dexamethasone compared to the standard of care in hospitalized patients with COVID-19 in a time horizon of one year from the perspective of health care. The findings of this study showed that if the costs associated with remdesivir were only related to hospitalization costs, remdesivir was cost-effective (dominant). Dexamethasone was also cost-effective with an ICER of USD 5,208 per QALY, and the simultaneous use of remdesivir and dexamethasone was the most favourable strategy (dominant). If remdesivir had an effect on reducing the mortality rate, its usefulness (the number of lives saved) would be three times higher than base cases (hazard mortality ratio = 0.91). If health care costs were not related to the length of the patient’s hospitalization, remdesivir was not cost-effective with an ICER of USD 384,412.8 per QALY. Dexamethasone was also cost-effective if the cost-effectiveness ratio of life saved was USD 313.79. This study concluded that remdesivir and dexamethasone were cost-effective. The results of the deterministic sensitivity analysis of remdesivir compared to the standard of care also showed that the most important variable affecting the results was the rate ratio for time to recovery. In this study, the most cost-effective strategy as the dominant option compared to standard treatment was remdesivir, which had the greatest cost savings [21].

### Cost-effectiveness results of the combination of remdesivir and baricitinib compared with remdesivir alone

The results of a cost-effectiveness study comparing the combination of baricitinib and remdesivir with remdesivir alone in hospitalized patients with COVID-19 in the United States showed that in the long-term time horizon the combination of these two drugs was cost-effective (cost-effectiveness ratios = 22,334 per QALY and 17,858 per life year gained). In all hospitalized patients, the combination of baricitinib and remdesivir compared to remdesivir reduced the total hospital expenses by USD 1778 per patient and reimbursement by up to USD 1526, and it increased QALY by 0.0018 and survival in hospitalized patients by 2.7% [24]. The results of a cost-effectiveness study into adding baricitinib to the standard of care (systemic corticosteroids and remdesivir) in the United States showed that the addition of baricitinib led to a QALYs gain of 0.6703 and LYs gain of 0.837 compared to the standard of care. The addition of baricitinib increased survival by 5.1% and decreased the use of mechanical ventilation by 1.6%. The results of deterministic sensitivity analysis showed that the most important variables influencing the results of the study were lifetime health care costs among recovered patients, followed by progress towards mechanical ventilation during hospitalization. The results of probabilistic sensitivity analysis showed that on the willingness-to-pay threshold of USD 50,000 per QALY with a probability of 96.5%, adding baricitinib to the standard of care was cost-effective. [25].

## Discussion

We conducted a systematic review of the cost-effectiveness of remdesivir for the treatment of hospitalized patients with COVID-19. After an extensive literature review, we identified 12 studies, which were of high quality.

Among the nine studies that performed the cost-utility analysis of remdesivir compared with standard treatment, five studies reported the cost-effectiveness of remdesivir [11, 15, 21–23]. A study conducted in Turkey showed that treatment with remdesivir saved costs compared to standard treatment, and remdesivir left more empty beds available in the hospital by shortening the length of stay and reducing the need for ventilation. In this study, real-world data from a hospital in Turkey and published data were used to estimate the effectiveness of remdesivir. This study used hydroxychloroquine, favipiravir, and/or dexamethasone as the standard treatment for severe pneumonia in hospitalized patients, according to Turkish treatment protocol at that time. This study considered four states of an economic model: general world-supplemental oxygen, ICU-supplemental oxygen, ICU-mechanical ventilation, and death. The study was conducted from the perspective of the payer and in the time horizon of a COVID-19 episode, and the discount rate of costs and benefits was not included in the study. As a result of the ACTT-1 trial, the length of stay and disutility of care were estimated for the remdesivir group, and the real-world results were used to estimate the length of stay and disutility of care for the standard group [13, 15]. Furthermore, the results of the cost-effectiveness study of 5-day remdesivir treatment for patients with severe COVID-19 compared to standard treatment in China showed that remdesivir, in addition to accelerating the recovery of patients, can free up hospital resources, which may have indirect benefits for other patients requiring hospital beds, thereby strengthening the capacity of health care facilities. Another point in this regard is the fact that hospital beds are scarce resources in many countries and fill up quickly when an epidemic escalates. Therefore, the use of a drug that allows us to free up hospital beds and manpower should be recommended even if it has no effect on mortality. This study was conducted from the perspective of the Chinese health system and on a time horizon of 55 days. The standard of care in this study included lopinavir, ritonavir, ribavirin, arbidol, chloroquine phosphate, hydroxychloroquine, and glucocorticoids. The states of the Markov model in this study were three states of mild, moderate and severe COVID-19 disease. Disutilities associated with mild infections have been estimated based on the 2010 Global Burden of Disease study. Furthermore, the disutilities of moderate and severe disease conditions have been estimated based on Chinese studies of severe respiratory infections and influenza outpatients. This study also assumes that the costs of the treatment arm are equivalent to those in high-income countries other than the United States [22]. In a study conducted in England, it was also indicated that remdesivir reduced recovery time, but this drug was cost-effective if it prevented death, and it was more cost-effective in COVID-19 patients with low-flow oxygen than those who had high-flow oxygen or who did not use non-invasive ventilation. This study was conducted from the perspective of the payer and on a lifetime horizon. The standard of care in this study was tocilizumab and sarilumab with or without corticosteroids. Furthermore, this study’s Markov model included three conditions: discharge from the hospital and survival, hospitalization with or without COVID-19, and death from any cause (COVID-19 or any other causes). The time when patients died in the standard care arm was also determined based on the RECOVERY trial [31]. Using the published data of the SOLIDARITY trial, the effects of treatment until discharge were analyzed [26, 32]. Adjusted death rates for the general population were taken from the 2017–2019 life tables for England and Wales. Moreover, the utility values were adjusted based on age and co-morbidities [26]. In addition, in a study conducted in South Africa, treatment with dexamethasone in both patients requiring mechanical ventilation and patients who did not require ventilation was predicted to prevent 689 deaths but increase treatment costs by USD 159,000 in comparison with standard treatment. The study assumed that 42% of the patients in the intensive care unit (ICU) required mechanical ventilation based on hospitalization data and guidelines for treating COVID-19 in South Africa, but 58% of them did not (if they required supplemental oxygen). However, remdesivir for patients who did not require ventilation and dexamethasone for patients who required ventilation prevented 408 deaths compared with standard treatment, resulting in a saving of 15 million dollars. The results of this study support previous findings on the effect of remdesivir on mortality by reducing the length of stay in the ICU and treating more COVID-19 patients in the ICU. The cost savings are mainly due to the reduction in the length of stay in the ICU. In this study, it was reported that the effectiveness of remdesivir and dexamethasone in preventing mortality may be influenced by several factors such as the time of treatment commencement after the onset of symptoms, age, comorbidities, potential side effects, and use of other medications.[23].

The results of an American study have revealed that if remdesivir improves patient survival in very severe COVID-19 patients who have a high risk of clinical outcomes and high health care costs and when remdesivir significantly improves the recovery time of patients and its impact on improving the patient’s quality of life is reflected, the possibility of cost-effectiveness of remdesivir will increase. If remdesivir is used in a population with less severe disease, its potential benefits to prevent mortality and its likely cost-effectiveness are reduced. This study was conducted from the perspective of the health system and over a lifetime, and it used the ACTT-1 trial and RECOVERY to estimate the probability of death from COVID-19 [13, 28, 31]. Additionally, the disutilities of disease states were estimated by using published studies, and the costs or disutilities associated with COVID-19 after discharge were not included [28]. The results of a research study in the United States showed that the use of dexamethasone for all patients was the most cost-effective strategy for the treatment of moderate and severe COVID-19 infections with a cost of USD 980.84/QALY per person per year compared to standard treatment. Remdesivir based strategies were all more expensive than other strategies in the base case. This study reported that dexamethasone emerged as the most cost-effective management method for all patients with moderate to severe COVID-19, and it was favorable for severe infections with a lower willingness-to-pay threshold. The study was conducted from the payer perspective over a 12-month period. In this study, it was assumed that there would be no progressing conditions after 28 days that could significantly impact patients’ quality of life. Moreover, the utilities of health conditions in this study were taken from published studies of patients diagnosed with H1N1 and influenza [14]. The cost of remdesivir may decrease once the generic version becomes available. This issue can significantly affect the cost-effectiveness of remdesivir. For example, it has been demonstrated that if the price of 100 mg vial of remdesivir is lowered to less than GBP 18.6, remdesivir will remain cost-effective, regardless of whether it reduces mortality in patients with COVID-19 [28].

All the studies included in the present review reported the cost-effectiveness of the combination of remdesivir and baricitinib compared to remdesivir alone [24, 25]. For example, the results of a cost-effectiveness study comparing the combination of baricitinib and remdesivir with remdesivir alone in hospitalized patients with COVID-19 in the United States showed that the combination of these two drugs was cost-effective on the long-term horizon. The study was conducted from the perspective of the payer and the provider over their lifetime. The COV-BARRIER trial results were used in this study to estimate effective treatments [25, 33]. Having fewer patients who required mechanical ventilation reduced inpatient costs since these patients are the most resource-intensive and costly to treat. Also, due to the higher survival of the combination of baricitinib and remdesivir compared to remdesivir alone, most of the difference in costs and QALYs was caused by increased survival (higher survival of the combination of baricitinib and Remdesivir). From the perspective of the hospital, the findings of this study showed that the combination treatment of baricitinib and remdesivir reduced total hospital costs compared to Remdesivir alone, primarily by reducing the proportion of patients requiring mechanical ventilation. Treatment with the combination of remdesivir and baricitinib leads to a reduction in hospital costs, which is estimated to be greater than the reduction in reimbursements received, resulting in net cost savings. In addition, from the perspective of the hospital, the combination of remdesivir and baricitinib compared to remdesivir alone shows a dominant option of cost-effectiveness (more benefits and lower costs) in the treatment of COVID-19 patients [25].

As part of the economic evaluation analysis, costs and benefits are evaluated from the perspective considered. Treatment and management of diseases are subject to third-party payer costs, which are incurred by third-party payers. Health care systems cover every medical expense, regardless of who pays for it. A health care system perspective includes out-of-pocket costs for patients and differs from the payer perspective [20, 34].

The effect of remdesivir on reducing the days of hospitalization is still the subject of debate since there are different results in other RCTs such as the WHO solidarity trial [21, 32]. According to the studies conducted, remdesivir may make hospitalization shorter [35, 36] or longer [37]. Moreover, at present, none of the cost-effectiveness models has considered the long-term consequences of COVID-19 due to the lack of data and the existing dynamic situation. There is still uncertainty regarding long-term mortality, side effects of treatments, and recovery time.

Our study has several limitations. As most of the studies included in the present review were conducted in the United States, our results are likely to be generalizable to high-income countries. The cost-effectiveness of remdesivir in low-income countries remains unknown. On the other hand, the results were limited to articles published in English, which, we believe, is a potential limitation of our systematic review. Further, the perspectives, data sources, and time horizons of the studies were different, and as a result, it is difficult to generalize the findings of the present study to other settings. Currently, there is no research on the cost-effectiveness of remdesivir for mild/moderate high-risk cases in outpatient settings. It is recommended to conduct further research on the cost-effectiveness of remdesivir in outpatient settings for mild/moderate high-risk cases.

## Conclusions

On the basis of the results of the present study, remdesivir was found to be cost-effective in comparison with the standard of care in China, Turkey, and South Africa. Studies conducted in the United States showed conflicting results (two studies indicated that remdesivir was cost-effective, and three studies showed that it was not cost-effective). Additionally, the study conducted in England showed that remdesivir could be cost-effective if it reduced mortality in patients with COVID-19. Moreover, the therapeutic strategy of combining remdesivir with Barcitinib was cost-effective compared to remdesivir alone. However, the cost-effectiveness of remdesivir in low-income countries remains unknown. More studies are required to determine how cost-effective this drug is.

## Supplementary Information

**Additional file 1.** PRISMA_2020_checklist.

**Additional file 2.** AMSTAR checklist.

**Additional file 3: Table S1.** Search strategy of databases.

## Data Availability

All data generated or analyzed during this study are included in this published article.

## Abbreviations

CEA: Cost-effectiveness analysis
CUA: Cost-utility analysis
QHES: Quality of Health Economic Studies
ECMO: Extracorporeal membrane oxygenation
DALYs: Disability-adjusted life years
LFO: Low-flow oxygen
HFO: High-flow oxygen
NIV: Noninvasive ventilation
ICER: Incremental cost-effectiveness ratios
QALY: Quality-adjusted life years
WTP: Willingness-to-pay
USD: United States dollar
GBP: British pound sterling
