# Supplementary material for: Cost-effectiveness of remdesivir for the treatment of hospitalized patients with COVID-19: a systematic review
Source: Infect Dis Poverty. 2023 Apr 20;12:39. doi: 10.1186/s40249-023-01092-1 (PMC10116457; doi:10.1186/s40249-023-01092-1)
Supplement: Supplementary file 3 — Additional file 3: Table S1. Search strategy of databases. [file 40249_2023_1092_MOESM3_ESM.docx]

Additional file 3: Table S1: Search strategy of databases

| Search strategy in PubMed |
| --- |
| (Cost[tiab] OR “Cost analysis”[tiab] OR (cost[tiab] AND analysis[tiab]) OR “cost comparison”[tiab] OR cost-effectiveness[tiab] OR “cost effectiveness”[tiab] OR cost-utility[tiab] OR “cost utility”[tiab] OR cost-benefit[tiab] OR “cost benefit”[tiab] OR "economic evaluation"[tiab] OR “economic evaluations”[tiab] OR “health resource allocation”[tiab] OR “health economic”[tiab] OR (economic[tiab] AND medical[tiab]) OR pharmacoeconomic[tiab] OR “decision analysis”[tiab] OR decision-analytic[tiab] OR economic*[tiab]) AND (covid-19[tiab] OR "2019 novel coronavirus disease"[tiab] OR "COVID 19"[tiab] OR "COVID-19 pandemic" [tiab] OR "SARS-CoV-2 infection"[tiab] OR "COVID-19 virus disease"[tiab] OR "2019 novel coronavirus infection"[tiab] OR "2019-nCoV infection"[tiab] OR "coronavirus disease 2019"[tiab] OR "coronavirus disease-19"[tiab] OR "2019-nCoV disease"[tiab] OR "COVID-19 virus infection"[tiab]) AND (remdesivir[tiab] OR Veklury[tiab] OR redyx[tiab]) AND 2020/01/01:2022/05/25[dp] |
| Search strategy in Web of Science Core Collection |
| (TS=(Cost) OR TS= (“Cost analysis”) OR (TS= (cost) AND TS= (analysis)) OR TS= (“cost comparison”) OR TS=(cost-effectiveness) OR TS=("cost effectiveness") OR TS=(cost-utility) OR TS=("cost utility") OR TS=(cost-benefit) OR TS=("cost benefit") OR TS=("economic evaluation") OR TS= (economic evaluations) OR TS=("health resource allocation") OR TS=("Medical Economics") OR (TS=(economic) AND TS=(medical)) OR TS=("health economics") OR TS=(economic*) OR TS=(“decision analysis”) OR TS=(decision-analytic) OR TS=(pharmacoeconomic)) AND (TS=(remdesivir) OR TS=(Veklury) OR TS=(redyx)) AND (TS=(covid-19) OR TS=("2019 novel coronavirus disease") OR TS=("COVID 19") OR TS=("COVID-19 pandemic") OR TS=("SARS-CoV-2 infection") OR TS=("COVID-19 virus disease") OR TS=("2019 novel coronavirus infection") OR TS=("2019-nCoV infection") OR TS=("coronavirus disease 2019") OR TS=("coronavirus disease-19") OR TS=("2019-nCoV disease") OR TS=("COVID-19 virus infection")) AND PY=(2020-2022) |
| Search strategy in Scopus |
| (TITLE-ABS(Cost) OR TITLE-ABS (“Cost analysis”) OR (TITLE-ABS (cost) AND TITLE-ABS (analysis)) OR TITLE-ABS (“cost comparison”) OR TITLE-ABS(cost-effectiveness) OR TITLE-ABS("cost effectiveness") OR TITLE-ABS(cost-utility) OR TITLE-ABS("cost utility") OR TITLE-ABS(cost-benefit) OR TITLE-ABS("cost benefit") OR TITLE-ABS("economic evaluation") OR TITLE-ABS (economic evaluations) OR TITLE-ABS("health resource allocation") OR TITLE-ABS("Medical Economics") OR (TITLE-ABS(economic) AND TITLE-ABS(medical)) OR TITLE-ABS("health economics") OR TITLE-ABS(economic*) OR TITLE-ABS(“decision analysis”) OR TITLE-ABS(decision-analytic) OR TITLE-ABS(pharmacoeconomic)) AND (TITLE-ABS(remdesivir) OR TITLE-ABS(Veklury) OR TITLE-ABS(redyx)) AND (TITLE-ABS(covid-19) OR TITLE-ABS("2019 novel coronavirus disease") OR TITLE-ABS("COVID 19") OR TITLE-ABS("COVID-19 pandemic") OR TITLE-ABS("SARS-CoV-2 infection") OR TITLE-ABS("COVID-19 virus disease") OR TITLE-ABS("2019 novel coronavirus infection") OR TITLE-ABS("2019-nCoV infection") OR TITLE-ABS("coronavirus disease 2019") OR TITLE-ABS("coronavirus disease-19") OR TITLE-ABS("2019-nCoV disease") OR TITLE-ABS("COVID-19 virus infection")) AND (PUBYEAR > 2019 AND PUBYEAR < 2023) |
| Search strategy in Embase |
| (Cost:ab,ti OR “Cost analysis”:ab,ti OR (cost:ab,ti AND analysis:ab,ti) OR “cost comparison”:ab,ti OR cost-effectiveness:ab,ti OR "cost effectiveness":ab,ti OR cost-utility:ab,ti OR "cost utility":ab,ti OR cost-benefit:ab,ti OR "cost benefit":ab,ti OR "economic evaluation":ab,ti OR “economic evaluations”:ab,ti OR "health resource allocation":ab,ti OR "health economic":ab,ti OR (economic:ab,ti AND medical:ab,ti) OR economic*:ab,ti OR pharmacoeconomic:ab,ti OR "decision analysis":ab,ti OR decision-analytic:ab,ti ) AND (remdesivir:ab,ti OR Veklury:ab,ti OR redyx:ab,ti) AND (covid-19: ab,ti OR "2019 novel coronavirus disease":ab,ti OR "COVID 19":ab,ti OR "COVID-19 pandemic":ab,ti OR "SARS-CoV-2 infection":ab,ti OR "COVID-19 virus disease":ab,ti OR "2019 novel coronavirus infection":ab,ti OR "2019-nCoV infection":ab,ti OR "coronavirus disease 2019":ab,ti OR "coronavirus disease-19":ab,ti OR "2019-nCoV disease":ab,ti OR "COVID-19 virus infection":ab,ti) AND [2020-2022]/PY |
| Search strategy in Cochrane |
| (Cost:ab,ti OR “Cost analysis”:ab,ti OR (cost:ab,ti AND analysis:ab,ti) OR “cost comparison”:ab,ti OR cost-effectiveness:ab,ti OR "cost effectiveness":ab,ti OR cost-utility:ab,ti OR "cost utility":ab,ti OR cost-benefit:ab,ti OR "cost benefit":ab,ti OR "economic evaluation":ab,ti OR “economic evaluations”:ab,ti OR "health resource allocation":ab,ti OR "health economic":ab,ti OR (economic:ab,ti AND medical:ab,ti) OR economic*:ab,ti OR pharmacoeconomic:ab,ti OR "decision analysis":ab,ti OR decision-analytic:ab,ti ) AND (remdesivir:ab,ti OR Veklury:ab,ti OR redyx:ab,ti) AND (covid-19: ab,ti OR "2019 novel coronavirus disease":ab,ti OR "COVID 19":ab,ti OR "COVID-19 pandemic":ab,ti OR "SARS-CoV-2 infection":ab,ti OR "COVID-19 virus disease":ab,ti OR "2019 novel coronavirus infection":ab,ti OR "2019-nCoV infection":ab,ti OR "coronavirus disease 2019":ab,ti OR "coronavirus disease-19":ab,ti OR "2019-nCoV disease":ab,ti OR "COVID-19 virus infection":ab,ti) |
